# Endoscopic inguinal lymphadenectomy in penile cancer: case report and literature review

**DOI:** 10.3332/ecancer.2015.576

**Published:** 2015-10-05

**Authors:** Juan Carlos Astigueta Pérez, Milagros Abad-Licham, Eloy Silva, Edgar Yan, Hugo Álvarez, Folker Agreda, Mariela Pow-Sang

**Affiliations:** 1 Department of Oncological Urology, Regional Institute of Neoplastic Diseases, Trujillo, 13007, Peru; 2 Private Antenor Orrego University, Faculty of Medicine, Trujillo, 13007, Peru; 3 Department of Oncological Pathology, Regional Institute of Neoplastic Diseases, Trujillo, 13007, Peru; 4 Department of Oncological Urology, National Institute of Neoplastic Diseases, Lima, 1500, Peru

**Keywords:** penile cancer, inguinal lymphadenectomy, endoscopy

## Abstract

**Objectives:**

The objective was to submit our first experience in endoscopic inguinal lymphadenectomy (EIL), evaluate the feasibility of the procedure and carry out a review of the literature.

**Material and methods:**

A 41-year-old patient was diagnosed with penile cancer with squamous cell carcinoma pT2G1 pathology, with no palpable inguinal lymph nodes. A bilateral inguinal lymphadenectomy was performed with preservation of the saphenous vein, conventional left and endoscopic right procedures. The perioperative data is presented and that obtained is discussed in the literature.

**Results:**

The total time was 270 minutes, 180 for endoscopic and 90 for conventional procedures. Blood loss was minimal in both cases. Fifteen lymph nodes were dissected on the endoscopic side, and 17 in the conventional side, the latter with more pain and devitalised skin flap.

**Conclusions:**

EIL for penile cancer is feasible and there is less morbidity with an early recovery. The literature is not conclusive on the indication of EIL.

## Introduction

In western countries, primary penile cancer is rare, with an average incidence below 1/100,000 inhabitants in Europe and the United States [1]. The lowest incidence worldwide is reported in Israeli Jews (0.1/100,000 inhabitants) [1, 2]. On the contrary, in the non-western world, the highest incidence rates are found in underdeveloped countries such as Uganda (2.8 /100,000 inhabitants), and in some areas of Brazil (1.5–3.7/100,000 inhabitants) [3–5].

In our environment (Peru), it is not uncommon and represents a constant therapeutic challenge, both of the penile tumour and the presence and extent of lymphatic metastases; considering the latter as the most important prognostic factor [6, 7].

In recent years, the introduction of minimal invasive techniques has allowed a more rapid recovery, less postoperative pain and in the case of the inguinal lymphadenectomy (IL) a lower morbidity related to the procedure, as well as a better display, recognition, and dissection of the anatomical structures [3, 8, 9].

Up until now, we have done eight EILs on patients diagnosed with penile cancer. We present a step-by-step technique applied in our first case with a follow-up to 24 months, and we also discuss the results and review the literature.

## Case report

The case is of a 41-year-old patient who had a total amputation of his penis with a pT2G1 squamous cell carcinoma pathological report. During the physical examination no palpable inguinal lymph nodes were found and the scar from the penile surgery was found to be in good condition. Studies for the extension of the disease were negative for metastasis. With these results in July 2012 he was subjected to: 1) Right endoscopic inguinal lymphadenectomy, and 2) a left conventional inguinal lymphadenectomy, both with conservation of the saphenous vein.

The pre-operative preparation was similar to that practised in conventional surgery, shaving the surgical area, low-molecular-weight heparin the day before, and antibiotic prophylaxis.

**Figure 1.**
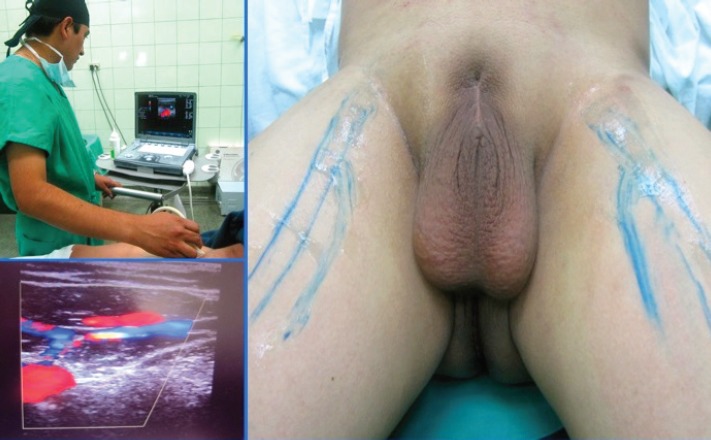
Doppler ultrasound of inguinofemoral region with the location of femoral vessels, the venous arch and saphenous vein, and marking of them on the skin.

Before surgery was carried out, the femoral vessels, venous arch, and the saphenous vein (Figure 1) were located and marked on the skin, with the help of Doppler ultrasound.

### Surgical technique

**Figure 2.**
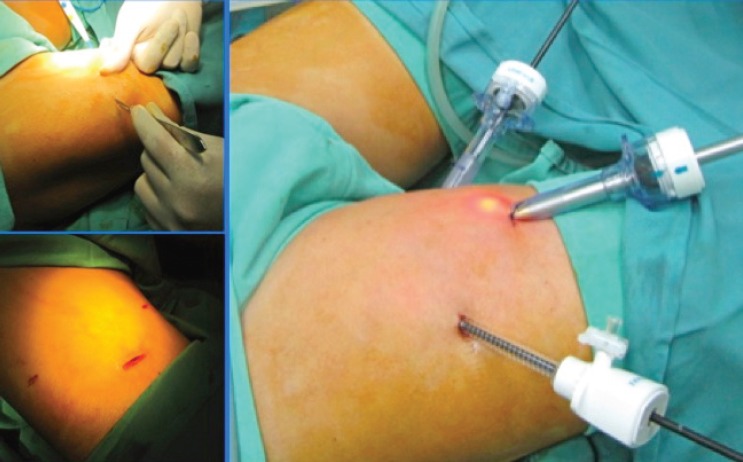
Trocar placement, two 10 mm (medial and central) and one 5 mm (lateral).

**Figure 3.**
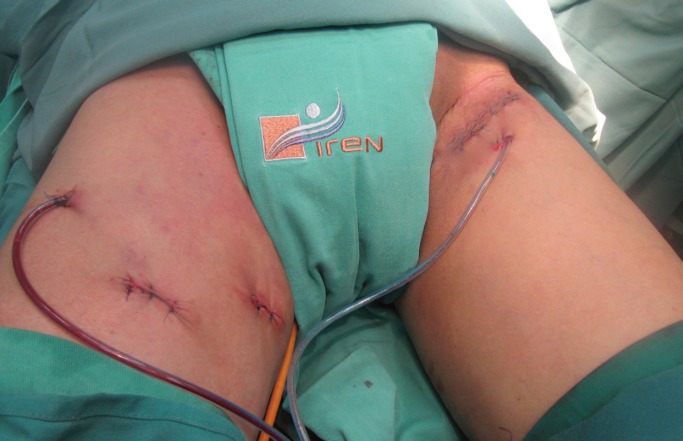
Surgical wounds of endoscopic inguinal lymphadenectomy (right) and conventional (left).

1. The tower with the monitor was placed near the head of the patient’s bed. The head surgeon was on the side of the surgical area and the assistant in front.
2. The patient under the effect of the anaesthesia was put into supine position with his lower limbs fixed into abduction, external rotation, and his knees bent.
3. Asepsis, antisepsis, placing a Foley catheter number 18 in neomeatus, and placing of sterile fields were accomplished.
4. Anatomically the femoral triangle was defined taking as limits the (top) inguinal ligament, the (lateral) sartorial muscle, and the long (medial) adductor muscle.
5. The first horizontal incision of 10 mm to 1 cm was made below the lower vertex, at the junction of the adductor muscles and long adductor, digitally developing a dissection plane by placing a 10 mm trocar through which the 0 degree optic was inserted and CO_2_ introduced to an initial 15 mm Hg pressure.
6. Orientated by transillumination the second and third incisions were carried out, triangulated to 8 cm of the first, in medial and lateral direction inserting 10 and 5 mm trocars respectively (Figure 2). The CO_2_ pressure is set at 10 mm Hg.
7. The dissection of the area was completed respecting the structural limits of the femoral triangle.
8. The femoral vessels and the vein arch was localised and dissected, the saphenous vein was dissected and other branches of the arch identified, all of which were clipped and sectioned. The dissection clamp, the angle, scissors, the sealed haemostatic clip (Ligasure®), and polymer clips (Hem-o-lok®) were used to acheive this.
9. During a second time, we located the dissection plane at the lower vertex level, between the superficial and deep subcutaneous cellular tissue (Scarpa’s fascia), and in a retrograde direction the resection of the tissue with the superficial and deep lymph nodes were completed. Whenever significant lymph or blood vessels were found during dissection, clips were applied.
10. Upon completion of the resection stage, the tissues were withdrawn in a bag through the first trocar wound.
11. The haemostasis was verified reducing the CO_2_ pressure to 5 mm Hg.
12. Tubular drainage closed to negative pressure (Hemosuc®) was inserted by the 5 mm trocar incision.
13. Finally, the skin borders were placed face to face.
14. On the left side a conventional inguinal lymphadenectomy (Figure 3) was carried out.

The video can be seen at http://www.youtube.com/watch?v=EiIVbJTjR3E.

## Results

Total surgical time was 270 minutes, 180 for the endoscopic procedure, and 90 for the conventional one. Blood loss was minimal on both sides.

There were no intra or postoperative endoscopic complications, the drainage tube was removed with a lower production than 50 cc. on the eighth day. Whereas on the conventional side, there was moderate pain and partial necrosis observed on the flap edges. Drainage was maintained until the 12th postoperative day when he was released from the hospital.

The anatomical and pathological study reported 15 nodes on the right-hand side (endoscopic) and 17 on the left (conventional), none of which had metastasis.

**Figure 4.**
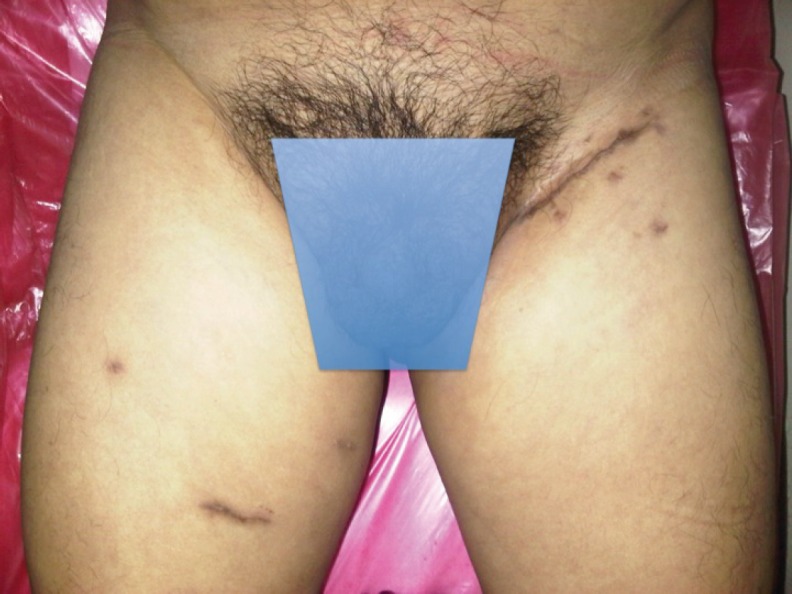
Bilateral inguinal lymphadenectomy cosmetic result.

During the 24 month monitoring no recurrence or progression of the disease occurred and the cosmetic results can be seen in Figure 4.

## Discussion

Squamous cell penile carcinoma is not a very common malignant disease, which substantially limits the value of epidemiological studies and understanding risk factors [1–5, 7]. The natural history of the disease starts with a physically and psychologically mutilating evolution with very poor therapeutic results when there is metastasis [2, 5]. Generally, patients usually die within two years of diagnosis of the primary lesion without treatment, caused by complications because of the uncontrollable locoregional or distant growth of metastases [5–7].

Currently, the dilemma for the urologist is the treatment of patients with non-palpable inguinal nodes which are approximately 20% of micrometastases. Considering that the presence of nodal metastasis is one of the major determinants of mortality, the LI indication can be performed in a prophylactic [1, 2, 6, 7] capacity.

According to different publications, patients with a negative clinical assessment and anatomopathological diagnosis of penile carcinoma above T1 G2 and/or lymphovascular infiltration are recommended to be treated with LI and probable pelvic lymphadenectomy, according to pathological results of the first [1–3].

Surgery is the foundation of the treatment of this pathology [1–3, 8], however, LI is not without complications and more than 50% of the cases present with lymph oedema, lymphocele, lymphorrhoea, infection, wound dehiscence, haemorrhage, haematoma, flap necrosis, deep vein thrombosis, thrombophlebitis, and even a 1–3% mortality rate related to surgery. In 1988 Catalonia published the modified LI technique, which has shown a decrease in morbidity and mortality over time with no negative effects on oncological results [2, 8].

In 2002, Ian Thompson, from the University of Texas, conceived the idea of endoscopic access for lymphadenectomy in penile cancer [2, 9], and Bishoff and collaborators reported two cases in corpses, and subsequently one in a living patient, in 2003. This surgery was not completed because of the nodes being fixed to the femoral vessels [10]. Based on these reports, the experiences in other specialities [11, 12], and after some modifications to the procedure described by Bishoff, the first case in the clinical urology scenario was successfully operated on in the Sao Paulo ABC Faculty of Medicine—Brazil by Tobias-Machado and collaborators [13, 14]. Another pioneer group in Latin America was led by René Sotelo from Venezuela, who in 2007 reported the results of 14 EILs on eight patients with squamous cell carcinoma pT2 [9].

There are currently several publications, basically case reports and small series, as well as some revisions mainly based on the Brazilian experience, where it is suggested and/or concluded that with the endoscopic technique there are fewer complications in comparison to conventional surgery, this is because of: 1) Less mechanical trauma produced by the retraction, 2) minimum use of electrocautery, 3) small incisions, which allow a better conservation of blood flow and lymphatic drainage of the skin, 4) no flap rotation of the sartorial muscle, and 5) easy identification of lymphatic vessels by magnification and control [1, 3, 9, 13–22].

This technique is not innocuous and there is a possibility of complications such as hypercarbia or postoperative pain; the first the same as in the laparoscopic procedures can easily be handled by the anaesthesiologist and the second with oral analgesics [16]. Sotelo and collaborators reported in their series of 14 EILs, three lymphoceles (23%); the Tobias-Machado group reported 20% of complications in 20 EILs; and Master in his series of 41 EILs, reported a total of 11 (27%) minor complications, and 6 (14.6%) greater complications [9, 15, 20].

Since 2007 there have been cases reported of EIL assisted by robot [23–25]; also with this technique, results of a phase I prospective study on patients with cancer of the penis T1-3N0 have been published, concluding that the inguinal dissection is adequate and should continue with the next phase to formally determine the incidence and types of complications, as well as the long term oncological effectiveness [26].

In 2013 the feasibility was reported of simultaneously carrying out the EIL with a reduction in operation time and the benefits already described as for the minimally invasive technique [27, 28].

In Peru penile cancer is not uncommon and the majority of patients come from populated areas far away from large cities, mainly in the mountains and wilderness, usually places with a low economic and cultural level. We, encouraged by the experience gained in the laparoscopic technique have planned and worked on our first case more than five years ago and also later analysed the multiple experiences published.

As described and stated by Sotelo *et al* (2007), prior to surgery, we located the femoral vessels, the saphenous vein, and the inguinal nodes sonographically, allowing us to anatomically and spatially get our bearings in endoscopic access and thus take greater care in dissection of the structures, while conserving the saphenous vein in order to reduce morbidity related to the procedure [9]. The time of the operation was 180 minutes, similar to those presented by other authors in their initial experiences with times between 120 and 180 minutes [14, 17–22]. There were no complications on the endoscopic side and results for the number of lymph nodes obtained were within what was expected.

We have found no reports of this technique in Peru, when reviewing the literature. To date we have performed eight procedures noting evolution in the technique and a reduction in surgical time with favourable cosmetic and oncological results that will be assessed in time.

## Conclusions

EIL to treat penile cancer is technically feasible, allowing adequate recognition of resection area limits and structures within it. There is a need for a good knowledge of the anatomy and its variants as well as experiences from laparoscopic surgery.

It is early to conclude that the EIL can be considered as standard in penile cancer with non-palpable lymph nodes, but it is evident by the many experiences reported, that it is an *alternative technique*, which has a lower morbidity with a speedy recovery when compared to the conventional technique.

More experiences are needed in this and other pathologies which use EIL. This will help in better evaluation of oncological results over time. One option or step towards it would be recording the management of information with an international cooperation database.

The video of this “Endoscopic Inguinal Lymphadenectomy in Penile Cancer,” surgery was presented in October 2013 in the XXXII American Confederation of Urology Congress, where it won First place in the Video Session: http://www.youtube.com/watch?v=EiIVbJTjR3E.

## References

[1] Pizzocaro G (2010). UAE Penile Cancer Guidelines 2009. Eur Urol.

[2] Pompeo AC, Heyns C, Abrams P (2009).

[3] Tobias-Machado M, Faria EF, Seabra DD, Machado RD (2009). Linfadenectomia Inguinal Videoendoscopica (Video-endoscopic inguinal lymphadenectomy).

[4] Pow-Sang M, Astigueta J (2009). HPV infection and the risk of penile cancer. J Andrological Sciences.

[5] Pow-Sang M (2010). Epidemiology and natural history of penile cancer. Urology.

[6] Slaton JW (2001). Tumor stage, vascular invasion and the percentage of poorly differentiated cancer: independent prognosticators for inguinal lymph node metastasis in penile squamous cancer. J Urol.

[7] Novara G (2007). Prognostic factors in squamous cell carcinoma of the penis. Nat Clin Pract Urol.

[8] Spiess PE, Hernandez MS, Pettaway CA (2009). Contemporary inguinal lymph node dissection: minimizing complications. World J Urol.

[9] Sotelo R (2007). Endoscopic lymphadenectomy for penile carcinoma. J Endourol.

[10] Bishoff JA (2003). Endoscopy subcutaneous modified inguinal lymph node dissection (ESMIL) for squamous cell carcinoma of the penis. J Urol.

[11] Folliguet TA (1998). Endoscopic saphenous vein harvesting versus ‘open’ technique. A prospective study. Eur J Cardiothorac Surg.

[12] Avrahami R (1998). Minimally invasive surgery for axillary dissection. Cadaveric feasibility study. Surg Endosc.

[13] Machado MT (2005). Comparative study between videoendoscopic radical inguinal lymphadenectomy (VEIL) and standard open lymphadenectomy for penile cancer: preliminary surgical and oncological results. J Urol.

[14] Tobias-Machado M (2006). Video endoscopic inguinal lymphadenectomy (VEIL): initial case report and comparison with open radical procedure. Arch Esp Urol.

[15] Tobias-Machado M (2008). Can video endoscopic inguinal lymphadenectomy achieve a lower morbidity than open lymph node dissection in penile cancer patients?. J Endourol.

[16] Tobias-Machado M (2009). 5-years experience with Video Endoscopic Inguinal Lymphadenectomy (VEIL): learning curve and technical variations of a new procedure. J Androl Sci.

[17] Master V (2009). Leg endoscopic groin lymphadenectomy (LEG Procedure): step-by-step approach to a straightforward technique. Eur Urol.

[18] Navarro M (2011). Linfadenectomia inguinal video asistida: experiencia inicial y resultados (assisted inguinal lymphadenectomy video: initial experience and results). Rev Chil Urol.

[19] Hernandez V (2011). Linfadenectomía inguinal video endoscópica en cáncer de pene. (Video endoscopic Inguinal lymphadenectomy in penile cancer). Aspectos técnicos (Technical aspects). Rev Mex Urol.

[20] Master V (2012). Minimally invasive inguinal lymphadenectomy via endoscopic groin dissection: comprehensive assessment of immediate and long-term complications. J Urol.

[21] Pahwa H (2013). Video endoscopic inguinal lymphadenectomy (VEIL) - a prospective critical perioperative assessment of feasibility and morbidity with points of technique in penile carcinoma. World J Surg Oncol.

[22] Zhou XL (2013). Endoscopic inguinal lymphadenectomy for penile carcinoma and genital malignancy: a preliminary report. J Endourol.

[23] Josephson DY, Jacobsohn KM, Link BA, Wilson TG (2009). Robotic-assisted endoscopic inguinal lymphadenectomy. Urology.

[24] Dogra P, Kumar A, Singh P (2011). Robotic-assisted inguinal lymph node dissection: a preliminary report. Indian J Urol.

[25] Sotelo R (2013). Robotic bilateral inguinal lymphadenectomy in penile cancer, development of a technique without robot repositioning: a case report. Ecancermedicalscience.

[26] Matin S (2013). Phase 1 prospective evaluation of the oncological adequacy of robotic assisted video-endoscopic inguinal lymphadenectomy in patients with penile carcinoma. BJU Int.

[27] Pompeo A (2013). Extending boundaries in minimally invasive procedures with simultaneous bilateral video endoscopic inguinal lymphadenectomy (veil) for penile cancer: initial experience and surgical considerations. Int Braz J Urol.

[28] Herrel LA (2012). Bilateral endoscopic inguinofemoral lymphadenectomy using simultaneous carbon dioxide insufflation: an initial report of a novel approach. Can J Urol.

